# Patient Satisfaction with CAD/CAM 3D-Printed Complete Dentures: A Systematic Analysis of the Clinical Studies

**DOI:** 10.3390/healthcare13040388

**Published:** 2025-02-11

**Authors:** Hanan N. Alotaibi

**Affiliations:** Department of Prosthetic Dental Sciences, College of Dentistry, King Saud University, Riyadh 11433, Saudi Arabia; haalotaibi@ksu.edu.sa

**Keywords:** 3D printing, CAD/CAM, complete dentures, elderly population, edentulous patients, oral rehabilitation, patient satisfaction

## Abstract

**Objectives**: This systematic review compares computer-aided design and computer-aided manufacturing (CAD/CAM) 3D-printed complete dentures (CDs) with conventional ones in terms of patient satisfaction. **Methods**: The PRISMA (Preferred Reporting Items for Systematic Reviews and Meta-Analyses) reporting criteria for systematic reviews were followed in conducting this systematic review. The study question was “What are the patient satisfaction outcomes of 3D-printed versus conventional CDs in edentulous patients?” according to the population, intervention, comparison, and outcome (PICO) framework. A comprehensive electronic search was conducted across three databases (PubMed/Medline, Web of Science core collection, and Scopus; last update: 18 August 2024) to obtain clinical trials that compared traditional and 3D-printed CDs. The retrieved articles were screened, their data were extracted, and their quality was evaluated. **Results**: The initial search retrieved 803 publications; 12 were chosen for a thorough review, and 5 of them—4 randomized cross-over studies and 1 randomized three-parallel arm study—met the requirements for this systematic review. One study showed significant differences in five of nine patient denture satisfaction domains, positively favoring the conventional CDs. Two studies showed non-significant differences in satisfaction domains between the conventional and 3D-printed groups, except for aesthetics and pronunciation. On the contrary, the satisfaction scores in two other studies showed no significant difference between the conventional and 3D-printed denture groups. **Conclusions**: The analysis of the included studies and evidence gathered demonstrates that CAD/CAM 3D-printed CDs seem to be comparable with conventional CDs in terms of overall patient satisfaction; however, 3D-printed CDs generate some concerns related to aesthetics and speech.

## 1. Introduction

Globally, the number of elderly individuals is steadily increasing owing to an increased life expectancy, rendering oral health concerns more critical for this group of individuals [1,2]. Losing all-natural teeth, or edentulism, is one of the most significant problems affecting older people. In addition to creating a considerable handicap, edentulism is associated with earlier mortality [3]. Removable complete dentures (CDs) have been a standard oral rehabilitation modality for completely edentulous patients for many years [4,5]. These prostheses satisfy a patient’s physical and social requirements but have not evolved much in recent years [6].

Polymethyl methacrylate (PMMA) is the most common material used in fabricating conventional complete dentures [7,8]. This material’s biocompatibility and aesthetic appeal and the ease with which it can be processed and repaired contribute to its growing patient acceptance [9,10]. On the contrary, polymerization shrinkage, the possibility of allergic reactions due to residual monomers, oral microbial colonization, the loss of radio-opacity, the deterioration of mechanical characteristics over time, and poor wear resistance are some of PMMA’s disadvantages [9,10]. Due to these issues, new materials and production methods are constantly being explored. In response to the challenges posed by conventional fabrication, CDs have been digitalized since the advent of computer-aided design and computer-aided manufacturing (CAD/CAM) technologies [9,10]. The refining of CAD/CAM technology in recent years, especially with respect to prosthodontics, has led to the development of various digital workflows and fabrication techniques that allow the production of CDs from start to finish using contemporary CAD-CAM workflows [11,12,13,14,15,16,17].

The two main techniques for the digital fabrication of CDs are additive and subtractive [18]. The denture base is milled, using the subtractive process, from a prepolymerized resin blank using a CAD design of the arch (stored as an STL file). The milling base is then attached to prefabricated or milled denture teeth [18]. Milled dentures have several drawbacks, such as requiring large manufacturing equipment, being associated with low productivity, having a time-consuming production process, being expensive, having a limited block size, and requiring a substantial amount of unused blank to be discarded [6,19,20]. The second technique for CAD/CAM denture fabrication is additive manufacturing (AM), frequently referred to as 3D printing or rapid prototyping (RP) [18]. The additive manufacturing process, which involves layer-by-layer laser beam scanning and polymerization of a methacrylate-based photocurable resin, is typically used to fabricate 3D-printed dentures [6,21,22]. While 3D printing is still primarily utilized to create trial dentures, most manufacturers use CAD/CAM milling to produce CDs commercially. However, this method is increasingly being used to fabricate definitive CDs [4]. The introduction of 3D-printed dentures was intended to address the drawbacks of the milling method, including by shorting manufacturing times, providing more predictable results, and offering cost-effectiveness [23]. The ability to produce multiple dentures at once, which is very cost-effective, is one of the additional benefits of 3D-printed dentures [15].

A primary objective of integrating CAD/CAM technologies into CD fabrication is to enhance patient access to oral healthcare [24,25]. CAD/CAM technologies can significantly increase elderly adults’ access to dental care. Patients’ treatment costs can be decreased by scheduling two to four denture treatment sessions rather than five, as is the case with conventional methods [21]. According to Lo Russo et al. [26], 3D-printed CDs are more profitable and cost-effective than milled base dentures. These developments greatly lower overall expenses, making advanced dental procedures more affordable and accessible to patients. Despite the upfront expenses, investing in digital technology can yield long-term financial gains [26].

Since patient-reported outcomes are more indicative than functional measures in terms of identifying treatment differences, they are especially significant when considering optional treatments [27]. Patient satisfaction with dentures is a considerable determinant of edentulous patients’ oral-health-related quality of life (OHRQoL). Furthermore, it is thought that patient satisfaction is more decisive for the success of CDs than denture quality [28]. In a swiftly aging population, there is a growing need for 3D-printed dentures since they are simple to fabricate and less expensive, and assessing patient satisfaction is crucial for clinical use [15]. Measuring satisfaction outcomes is simple and enables the direct measurement of patients’ feelings and thoughts regarding various prosthodontic treatment aspects [29,30]. A substantial quantity of data indicates that 65–90% of patients are satisfied with traditional complete dentures [31]. Despite these considerations, only a small number of patients seem to be dissatisfied.

Complete dentures fabricated using various methods should be indistinguishable, provide similar degrees of satisfaction, and have similar appearances and functions. However, as distinct materials, manufacturing processes, and polymerization techniques are employed in these two methods, there might be a difference for rational reasons [4]. In the past few years, a few systematic reviews have compared CAD/CAM CDs with conventional CDs in terms of patient satisfaction [32,33,34,35]. These studies have demonstrated that CAD/CAM CDs are comparable or superior to conventional dentures. However, these reviews have largely focused on milled dentures, and the outcome appears generalized. The reason behind this outcome is the limited availability of clinical studies evaluating the outcomes of comparisons of 3D-printed CDs with conventional CDs.

Positive patient experiences are also a major factor in the success of denture treatment. Incorporating patient perceptions into the finished dentures is essential for attaining successful outcomes. As with many healthcare procedures, the primary criterion for assessing denture care is patient-reported outcome measures (PROMs). In other words, it is unknown how patients view CAD/CAM dentures and whether they function well enough from their perspectives [21]. Previous studies evaluating 3D-printed and conventional denture materials regarding patient satisfaction have demonstrated diverse and inconclusive outcomes [5,15,16,23,36]. Thus, there is a need to compare CAD/CAM 3D-printed dentures to gold-standard conventional dentures concerning patient satisfaction, considering the increased use of 3D-printed CDs in clinical practice. To the best of the author’s knowledge, previous studies have largely focused on milled dentures, and the data related to 3D-printed dentures are limited. Consequently, this study aimed to review and analyze the clinical studies evaluating patients’ satisfaction with CAD/CAM 3D-printed dentures in comparison to conventional dentures.

## 2. Materials and Methods

### 2.1. Study Design

This study systematically analyzes clinical studies evaluating patients’ satisfaction with 3D-printed complete dentures. The research question was “What are the patient satisfaction outcome of 3D-printed versus conventional CDs in edentulous patients?”. This study compiled with the PRISMA (Preferred Reporting Items for Systematic Reviews and Meta-Analyses) guidelines for reporting systematic reviews, and the PRISMA checklist for this systematic review is provided in the Supplementary Materials. This review was not registered.

### 2.2. Eligibility Criteria

Before the literature-screening process was initiated, eligibility criteria were established based on PICOS (population, intervention, comparison, outcomes, and research design) domains (Table 1). Any clinical trial or observational or prospective study that assessed patients’ satisfaction with CAD/CAM 3D-printed CDs vs. conventional CDs published in English was included. Articles published in other languages were excluded due to a lack of resources (professional translators and funding) and time. Studies evaluating partially edentulous patients, retrospective studies, in vitro research, surveys and case reports, and other review articles were not included.

### 2.3. Literature Database and Search Strategy

An electronic search was conducted on three databases, namely, PubMed/Medline, Web of Science (WOS) core collections, and Scopus, to identify studies published in English that adhered to the eligibility criteria. The search was restricted from 1 January 2010 to 18 August 2024 in order to find the most recent articles available and ensure accurate information was obtained. The literature search was performed by the author and a full professor at the Department of prosthodontics at the same university. The literature search included identifying relevant keywords and search terms. The search terms or keywords used were as follows: patient satisfaction with 3D-printed complete denture OR OHRQoL of 3D-printed complete denture OR Patient satisfaction with rapid prototyped complete denture OR Patient related outcomes of 3D printed complete denture OR Patient related outcomes of rapid prototyped complete denture OR Patient related outcomes of CAD/CAM 3D printed complete denture OR Patient satisfaction with conventional and CAD/CAM 3D printed complete denture OR Patient satisfaction with conventional and CAD/CAM 3D printed overdenture OR OHRQoL of 3D-printed overdenture. In addition to the databases, a manual search was performed in Google scholar.

### 2.4. Process of Study Selection

A bibliographic reference management program (EndNote^®^, Clarivate Analytics, Philadelphia, PA, USA) was used to import all articles retrieved through databases and manual searches. The same program was used to identify and remove duplicates. The remaining articles were chosen according to the eligibility criteria after being vetted by reviewing the title and abstract. Full-text studies deemed pertinent to this study were manually retrieved and thoroughly reviewed. The final studies to be included were subsequently chosen using the inclusion and exclusion criteria. Article selection bias was eliminated via a clearly stated research question (PICOS), a relevant search strategy using databases, appropriately defined eligibility criteria, and thorough review of the selected articles by the reviewers.

### 2.5. Quality Assessment

Using the quality assessment tool produced by the National Heart, Lung, and Blood Institute (NHLBI) (accessible at https://www.nhlbi.nih.gov/health-topics/study-quality-assessment-tools, accessed on 3 August 2024), an evaluation of the methodological quality of the original articles was conducted. The criteria were assessed using the following answers: “Yes”, “No”, or “Other” (not reported, not applicable, or not determinable). Each study was rated based on the positive response factors: ≥75% = good, 50–75% = fair, and <50% = poor [37]. The evaluation was performed independently by the author and the professor. Another professor from the same department was consulted in case of a discrepancy, which was resolved with a consensus meeting.

## 3. Results

### 3.1. Outcome of Literature Search

**Figure 1:**
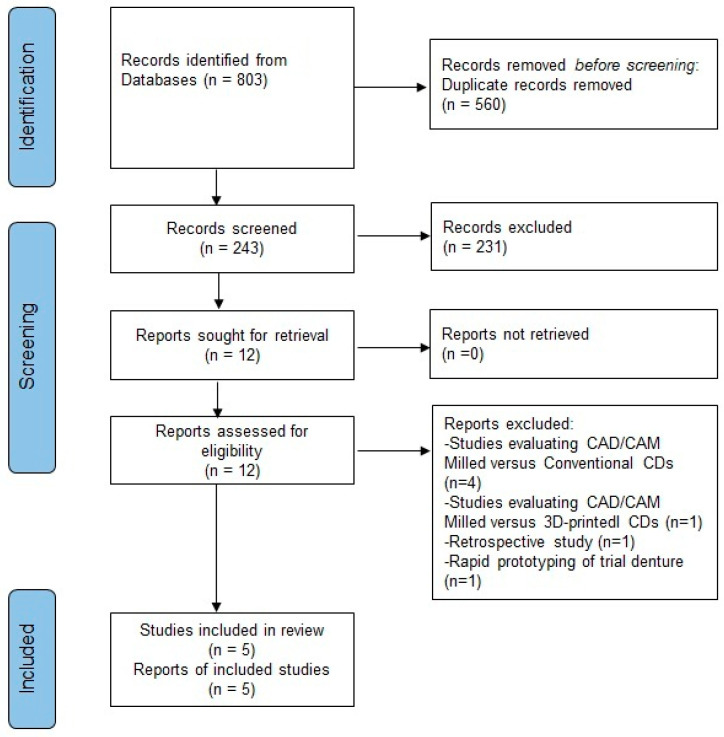
PRISMA flowchart.

Figure 1 shows a PRISMA flowchart showing the study process. Eight hundred and three articles were identified using a literature search of the three databases (Pubmed/Medline—485, Web of Science core collection—296, and Scopus—16) and a manual search using Google Scholar and a reference list (6). After deleting 560 duplicates, the remaining articles were reviewed independently by the author and the full professor to further eliminate 231 articles based on their titles and abstracts, leaving 12 for further review. The inter-reviewer agreement was calculated using percentage agreement, and the level of agreement was 98%, which implies perfect agreement between the reviewers. As per the plan for this study, another professor at the same department would have been consulted in case of any disagreement between the reviewers. The author and the professor read all 12 articles in their entirety for further analysis. Seven articles were excluded, among which five articles evaluated milled and conventional dentures [14,22,27,38], with no 3D-printed interventional groups; one evaluated milled dentures with 3D-printed groups [4]; one was a retrospective study [39]; and the other study evaluated a rapidly prototyped trial denture, not a final denture used in practice [36]. Finally, five articles (four randomized cross-over studies and one randomized three-arm parallel study) were included in this review by the two reviewers (the author and the professor) based on the inclusion criteria [5,7,15,16,23]. Table 2 shows the studies excluded along with the reasons for exclusion after reading the full texts.

### 3.2. Outcome of the Quality Assessment of the Included Studies

The NHLBI quality evaluation tool was used to assess the quality of the included original articles. The assessment outcomes of the studies were categorized as good (>75%; score: >10), fair (50–75%; score: 7–10), or poor (<50%; score: <6) [37]. Table 3 presents the quality assessment of the studies based on the assessment criteria. The quality of two of the studies was rated “good,” and three studies were rated as “fair”. Two of the four randomized cross-over studies were rated “good,” and two were “fair”. The quality of the three-arm parallel randomized study was rated “fair”. Overall, two studies were rated good, which implies that these studies have the least risk of bias and study results that can be considered valid. The remaining three studies were rated fair, which implies that there may be some bias in these studies but not enough to render the results unreliable [40].

### 3.3. Characteristics of the Analyzed Studies

A detailed compilation encompassing the design, participant grouping and allocation, variables, intervention, results, and final conclusions of each study was made to enable a thorough review and analysis. However, a qualitative analysis was used because of the diversity of the included studies and the assessments of satisfaction. Table 4 presents the characteristics of the included studies. There were two studies from Egypt [5,7], two from Japan [15,23], and one from South Korea [16].

A total of 110 patients were enrolled in these clinical studies. The ages of the patients in these studies ranged from 45 to 90 years. Regarding gender, there were 20 female patients and 26 male patients in three studies [15,16,23]. However, no gender distributions were reported in the two studies from Egypt [5,7].

In three of the five studies, 3D-printed dentures were compared with conventional dentures [15,16,23]. One study compared 3D-printed and milled dentures with conventional dentures [5], and one study compared mandibular 3D-printed dentures with conventional overdentures [7].

### 3.4. Patient Satisfaction Evaluation Periods and Instruments

The patient satisfaction outcomes in these studies were assessed using various questionnaires and methods. Table 5 summarizes the evaluation period and measurement approaches in terms of patient satisfaction (Table 5). Four studies employed a visual analogue scale (VAS) to measure patient satisfaction [7,15,16,23], while one used a Likert scale to assess the domains and VAS for assessing general satisfaction [5]. Among the studies in which a VAS was used to determine satisfaction, one study just evaluated satisfaction by using anchor words, namely, “completely dissatisfied” and “completely satisfied”, without assessing any specific parameters [15]. The other studies in which VAS was used evaluated patient satisfaction regarding mastication, pain, retention, stability, comfort, esthetics, cleaning efficacy, speech, color and contours of the dentures, taste, attachment to gum tissue, and overall satisfaction. Regarding the assessment period for patient satisfaction, two studies assessed patient satisfaction 1 month after denture delivery [15,16], one study evaluated it at baseline and after denture adjustment [23], one study assessed it 3 months after provision [8], and one study, by Heikal et al. [5], assessed satisfaction 2 weeks and 3 and 6 months after provision. This study presents the qualitative data rather than quantitative patient satisfaction scores since the assessment methods and periods varied from study to study. For research with comparable results, assessing similar outcomes, and possessing obtainable numerical data, a quantitative analysis is appropriate. Accordingly, a meta-analysis was not possible in this systematic review because of the significant disparities between the studies that were included in the analysis.

### 3.5. Patient Satisfaction Outcomes

Among the analyzed studies, the study by Ohara et al. [23] showed significant differences in five of nine patient denture satisfaction domains in favor of conventional CDs. The studies by Nabil et al. [7] and Kang et al. [16] showed non-significant differences between the domains of satisfaction between the conventional and 3D-printed groups, except for aesthetics and pronunciation, respectively. However, general satisfaction was non-significantly different between the two study groups. On the contrary, the satisfaction scores in the studies by Heikal et al. [5] and Iwaki et al. [15] showed no significant differences between the conventional and 3D-printed denture groups (Table 6).

## 4. Discussion

Complete edentulism is regarded as a rapidly increasing public health issue [43]. CAD/CAM CDs have been suggested as an alternative to dentures fabricated using the conventional method, especially when time and access to care are important factors. CAD/CAM technology can save resources by reducing chairside time and enabling virtual or online appointments [24,25]. Specifically, the same dentures can be refabricated using digital data when the patient is not present, which could be very beneficial, especially in the case of geriatric patients. Furthermore, patients may avoid the need for a clinical appointment by using the internet to obtain 3D images of their faces with their future dentures [21]. However, it is crucial to confirm whether dentures offer patients satisfaction or care comparable with or superior to conventional treatment before replacing the conventional CD fabrication method. Nevertheless, this study analyzed clinical studies to attain an answer to the question formulated as per the PICOS criteria: “What are the patient satisfaction outcome of 3D-printed versus conventional CDs in edentulous patients?”. The outcome of this review demonstrated that 3D-printed complete dentures were comparable in terms of patient satisfaction.

A substantial level of patient satisfaction should be the primary objective of any prosthodontic treatment provided to edentulous patients since it is a crucial factor in assessing the effectiveness and quality of care [31]. However, various objective and subjective elements may contribute to patient satisfaction. In this context, domains from patients’ perspectives such as mastication, pain, retention, stability, comfort, esthetics, cleaning efficacy, and speech play a significant role in determining a patient’s satisfaction with new dentures [7,16,23,44].

In the current study, 12 full-text studies were found and evaluated according to the eligibility criteria, which led to the exclusion of 7 articles. The remaining five articles were thoroughly assessed. The quality of two articles was rated “good”, and three studies were considered to have “fair” quality. Only one study by Ohara et al. [23] demonstrated that 3D-printed CDs were inferior to conventional CDs, while the other studies showed that both conventional and 3D-printed CD types were comparable in terms of patient satisfaction. Furthermore, when asked to choose between the two denture types, only 20% of the patients in Ohara et al.’s [23] study preferred 3D-printed dentures, while 80% of the patients preferred conventional dentures. This difference was largely related to the significant differences in the following satisfaction domains: phonetics, ease of cleaning, stability, comfort, and general satisfaction. The stable occlusal contacts and marginal seal at the denture borders may be the reason for the increased stability and comfort provided by CDs. The marginal seal of printed dentures may be inferior to that of conventional dentures because the former are designed to include the maximally extended imprint and fringe thickness, chosen arbitrarily [23]. Furthermore, patients are more satisfied with the conventional method compared to the digital methods, which do not involve definitive impressions, especially when a professional dentist fabricates complete dentures for an edentulous mandible [45].

In this review, a comparison of 3D-printed overdentures with conventional overdentures was also included for the analysis [7], as overdentures are regarded as the standard of care for completely edentulous patients according to the international consensus [46]. The outcome of the cited study showed that the conventional CDs’ aesthetics were better than those of 3D-printed dentures. Unlike conventional CDs, 3D-printed dentures received the lowest ratings, primarily in the tooth-related category (white appearance), compared to conventional dentures [47]. This could be explained by variations in the fabrication process. While 3D-printed artificial teeth are identical to resin teeth in terms of their physical characteristics aside from their single-color tone, prefabricated artificial teeth are more visually akin to natural teeth, with several visible areas of translucency and optical characteristics [7]. However, this drawback may soon be resolved owing to newer resins and more complex printers on the market [8].

Although Kang et al. [16] demonstrated that patients with 3D-printed dentures were equally as satisfied as the conventional denture group, the authors noted a significant difference in pronunciation between the groups, with better pronunciation in the conventional denture group. Notably, 3D-printed dentures are fabricated with a palatal thickness of 2.5 mm to maintain strength, whereas conventional dentures are made with a thickness roughly equal to that of a paraffin wax sheet, around 1.4 mm. This possibly explains why conventional dentures are associated with considerably greater phonetic satisfaction than 3D-printed dentures [23].

Among the studies analyzed, four were randomized cross-over studies. The primary characteristic that sets a crossover study apart from the standard parallel-group trial is that each patient acts as their own control [48]. Thus, the crossover design skirts issues pertaining to study and control group comparability concerning confounding characteristics (e.g., age and sex). Additionally, the crossover design has an advantage in terms of the statistical test’s power to verify the presence of a treatment effect [48]. On the contrary, factors like carryover should be assessed before examining the treatment effect in studies employing a crossover design since they significantly impact the treatment outcome [49]. Secondly, the washout phase must be sufficiently extended to completely rule out a carryover effect from one treatment period to the next. However, in the studies analyzed in this review, the lengthy washout period was not employed due to an ethical reason, namely, not wearing dentures for research purposes.

It might be argued that each patient’s level of satisfaction with complete dentures is unique, and a self-reported response style enables patients to voice their thoughts on their own [31]. The methods used to evaluate patient satisfaction differed considerably between the studies, ranging from questionnaires to VAS. Another important factor influencing patient satisfaction outcomes is the period of evaluation. The included studies had short evaluations of patient satisfaction: one week to six months after denture provision. The insignificant outcomes of the comparison of the conventional and 3D-printed dentures in most of the analyzed studies could be attributed to the short-term evaluations. One could argue that, in contrast to the short follow-up period, patient satisfaction could vary over longer follow-up periods [31]. Improvement in the domains linked to patient satisfaction after an intervention takes some time. It has been shown that patients wearing digital dentures experience sore spots, discomfort, painful mucosa, and chewing difficulty, even 6 months after the insertion of new dentures [50]. Prior research has demonstrated a significant enhancement in quality of life, a determinant of patient satisfaction, from six months to two years after CD provision [51,52,53]. The difference in the evaluation methods and period did not allow for the quantitative measurement of the satisfaction scores.

The relatively small number of clinical trials included in this systematic review is one of its limitations. Four of the five studies involved comparisons of conventional CDs versus 3D-printed CDs, and one study compared conventional and 3D-printed overdentures. Any inferences made from these articles have low data support because of the influence of cohort size, effect size, and significance level. The lack of clinical studies employing 3D-printed CDs could be related to the substantial in vitro research and clinical investigations comparing conventional and milled CDs. Therefore, in order to derive definite outcomes, an adequate number of methodologically planned RCTs with adequate sample sizes and sufficient power are required, in addition to a long-term satisfaction assessment of 3D-printed CDs. Furthermore, more studies are required from different parts of the world in order to attain a clearer understanding of global patient satisfaction regarding 3D-printed CDs.

## 5. Conclusions

Within the limitations of the study, the gathered evidence demonstrates that CAD/CAM 3D-printed CDs seem comparable to conventional CDs in terms of overall patient satisfaction; however, 3D-printed CDs generate some concerns related to aesthetics and pronunciation. Furthermore, 3D-printed CDs could be recommended as an alternative to conventional CDs for edentulous patients owing to their comparable levels of satisfaction, reduced costs, and the possibility of improved access to care for the elderly. Future studies should focus on analyzing the specific factors that affect patients’ satisfaction with digital dentures in addition to a long-term evaluation of patient satisfaction.

## Figures and Tables

**Table 1:** PICOS criteria for inclusion of studies.

PICOS Domain	Criteria
Population	Edentulous patients, aged >18 years and treated with CDs
Intervention	3D-printed CDs
Comparison	Conventionally fabricated CDs
Outcomes	Patient satisfaction
Study design	Randomized or non-randomized clinical trials, cross-sectional studies, observational clinical studies, and prospective clinical studies

**Table 2:** Studies excluded after reading full texts.

Articles Excluded	Exclusion Criterion
Inokoshi et al. (2012)	Rapidly prototyped trial dentures versus conventional trial dentures Final dentures were not evaluated [36]
Kattadiyil et al. (2015)	Conventional versus milled dentures No 3D-printed interventional denture group was evaluated [14]
Saponara et al. (2016)	Conventional versus milled dentures No 3D-printed interventional denture group was evaluated [38]
Kim et al. (2021)	Retrospective reporting of patient outcomes Patient satisfaction was not evaluated [39]
Srinivasan et al. (2021)	3D-printed versus milled dentures Conventional dentures were not evaluated [4]
Peroz et al. (2022)	Conventional versus milled dentures No 3D-printed interventional denture group was evaluated [22]
Zupancic et al. (2023)	Conventional versus milled dentures No 3D-printed interventional denture groups were evaluated [27]

**Table 3:** Quality Assessment of the included studies.

Author (Year)	Quality Assessment Criteria														Total Score (%)	Quality Rating
	1	2	3	4	5	6	7	8	9	10	11	12	13	14		
Ohara et al. (2022) [23]	Y	Y	Y	N	Y	Y	N	N	Y	Y	CD	Y	Y	Y	10/14 (71.4%)	Fair
Heikal et al. (2022) [5]	Y	Y	Y	N	Y	Y	NR	NR	Y	Y	CD	Y	Y	Y	10/14 (71.4%)	Fair
Nabil et al. (2024) [7]	Y	Y	Y	Y	Y	Y	N	N	Y	Y	CD	Y	Y	Y	11/14 (78.5%)	Good
Iwaki et al. (2024) [15]	Y	Y	Y	N	N	Y	Y	Y	Y	Y	N	Y	Y	Y	11/14 (78.5%)	Good
Kang et al. (2024) [16]	Y	Y	Y	N	N	NR	Y	Y	Y	Y	N	CD	Y	Y	9/14 (64.2%)	Fair

Quality ratings: poor <50%, fair 50–75%, and good >75%. Y, yes; N, no; CD, cannot determine; NR, not reported.

**Table 4:** Characteristics of the analyzed studies.

	Studies	Characteristics	Description
1	Ohara et al. (2022) [23]	Study design	Randomized cross-over clinical trial
		Aim	To compare patient satisfaction between conventional and 3D-printed CDs
		Ethical approval	Obtained
		Participants	Two groups (n = 10/group); age: 66–90 years
		Interventions	3D-printed CDs
		Comparison	Conventional CDs
		Blinding	No
		Washout period	Followed but not reported
2	Heikal et al. (2022) [5]	Study design	Randomized three-arm parallel clinical trial
		Aim	To assess different denture fabrication methods in terms of patient satisfaction and retention
		Ethical approval	Obtained
		Participants	Three groups (n = 16/group); age: 55 to 75 years
		Intervention	3D-printed and milled CDs
		Comparison	Conventional CDs
		Blinding	Single blind (patients)
		Washout period	Not applicable
3	Nabil et al. (2024) [7]	Study design	Randomized cross-over clinical trial
		Aim	To assess and compare masticatory performance and patient satisfaction for conventional and 3D-printed mandibular implant overdentures.
		Ethical approval	Obtained
		Participants	Two groups (n = 8/group); age: 45 to 60 years
		Intervention	3D-printed mandibular overdentures
		Comparison	Conventional mandibular overdentures
		Blinding	Not reported
		Washout period	Two-weeks
4	Iwaki et al. (2024) [15]	Study design	Randomized cross-over clinical trial
		Aim	To assess and compare QoL and patient satisfaction for conventional and 3D-printed CDs.
		Ethical approval	Obtained
		Participants	Two groups (n = 10/group); Age 65–84 years
		Intervention	3D-printed CDs
		Comparison	Conventional CDs
		Blinding	Single blind (patients)
		Washout period	No washout period
5	Kang et al. (2024) [16]	Study design	Randomized cross-over clinical trial
		Aim	To assess clinical performance and patient satisfaction for conventional and 3D-printed CDs.
		Ethical approval	Obtained
		Participants	Two groups (n = 4/group); age: 66 to 83 years
		Intervention	3D-printed CDs
		Comparison	Conventional CDs
		Blinding	Not reported
		Washout period	Followed but not reported

**Table 5:** Measurement instruments used to evaluate patient satisfaction.

Studies	Evaluation Period	Measurement Instrument
Ohara et al. (2022) [23]	Baseline and after denture adjustment	A 100 mm visual analogue scale (VAS) was used to quantify mastication, pain, retention, stability, comfort, esthetics, cleaning efficacy, speech, and overall satisfaction.
Heikal et al. (2022) [5]	2 weeks and 3 and 6 months after delivery	Satisfaction was evaluated as per Boerrigter et al.’s approach [41]. A questionnaire containing five main domains was used: (a) Functional complaints about the denture (13 questions) (b) Overall masticatory ability (6 questions) (c) Masticating ability for different types of food (3 questions) (d) The effects on one’s mental state and daily life (7 questions) (e) Overall denture satisfaction (9 questions) Each domain received a rating on a Likert scale, except the last domain, which received a rating on a VAS. The last two questions had yes or no answers.
Nabil et al. (2024) [7]	3 months after delivery	A questionnaire containing eight core items from the McGill Denture Satisfaction Questionnaire (MDSQ) [42] was used. The first item pertained to overall satisfaction with the current prosthesis, while the remaining seven items pertained to specific parameters such as comfort, esthetics, cleaning efficacy, speech, aesthetics, stability, chewing ability, and function. The responses were recorded on a 100 mm VAS.
Iwaki et al. (2024) [15]	1 month after delivery	Overall satisfaction was recorded on a 100 mm VAS with the anchor words “completely dissatisfied” and “completely satisfied”, and the response was converted to a score. However, the study did not assess specific parameters on satisfaction.
Kang et al. (2024) [16]	1 month after delivery	A 12-item questionnaire was answered on a 10 cm long VAS. The questions pertained to the color and contours of dentures, chewing ability, taste, speech, pain or discomfort, attachment to gum tissue, and individual satisfaction scores for maxillary and mandibular dentures.

**Table 6:** Patient satisfaction outcomes in the analyzed studies.

	Studies	Patient Satisfaction Outcomes
1	Ohara et al. (2022) [23]	Conventional dentures had significantly higher scores than 3D-printed dentures in terms of phonetic qualities, ease of cleaning, stability, comfort, and general satisfaction
2	Heikal et al. (2022) [5]	The 3D-printed dentures had non-significantly lower mean scores than conventional dentures
3	Nabil et al. (2024) [7]	Mean satisfaction scores were comparable between the overdenture groups, except in regard to the aesthetic scores, which were better for conventional overdentures.
4	Iwaki et al. (2024) [15]	The mean scores of patient satisfaction were not significantly different between the conventional and 3D-printed groups.
5	Kang et al. (2024) [16]	The mean scores evaluating satisfaction were not significantly different between the denture groups, except in regard to pronunciation, which was significantly better for conventional dentures.

## Data Availability

Not applicable.

## Supplementary Materials

The following supporting information can be downloaded at: https://www.mdpi.com/article/10.3390/healthcare13040388/s1, PRISMA 2020 Checklist [54].
